# Low morphology does not lower success after intrauterine insemination unless inseminating motile sperm count is low

**DOI:** 10.1371/journal.pone.0317521

**Published:** 2025-03-19

**Authors:** Heather Burks, Jennifer D. Peck, Karl R. Hansen, Julie Stoner, LaTasha B. Craig

**Affiliations:** 1 Department of Obstetrics and Gynecology, Section of Reproductive Endocrinology and Infertility, The University of Oklahoma College of Medicine, Oklahoma City, Oklahoma, United States of America; 2 Department of Biostatistics and Epidemiology, Hudson College of Public Health, The University of Oklahoma Health Sciences, Oklahoma City, Oklahoma, United States of America,; 3 Posthumous Contribution, Department of Biostatistics and Epidemiology, Hudson College of Public Health, The University of Oklahoma Health Sciences, Oklahoma City, Oklahoma, United States of America; Bangladesh Agricultural University, BANGLADESH

## Abstract

The objective of this study was to determine the relationship between strict morphology as assessed on the initial semen analysis during fertility workup and pregnancy rates after intrauterine insemination. This is a retrospective study of couples undergoing intrauterine insemination from 2007 to 2012. Couple characteristics and semen analysis parameters were recorded and evaluated. Risk ratios (RR) and 95% confidence intervals (95% CI) were calculated, accounting for within-couple (cluster) correlation among repeated intrauterine insemination cycles. Four hundred thirty-five women (average ±  standard deviation age 31.7 ±  4.8) undergoing 1,287 intrauterine insemination cycles were analyzed. Fecundability was not statistically different when low strict morphology (≤1% and 2-4%) was compared to the reference range of morphology > 14% [RR 0.99 (0.41-2.40) and 0.90 (0.48-1.70)]. Results were unchanged when adjusted for female characteristics, medication, and inseminating total motile sperm count [aRR 1.22 (0.51-2.93) and 1.00 (0.53-1.91)]. Evaluating combined effects of morphology with inseminating total motile sperm count, pregnancy rates among cycles with total motile count <  5 million and strict morphology ≤  4% normal were reduced when compared to cycles with total motile count > 20 million and morphology > 4% normal (RR 0.37, 95% CI 0.17-0.82). These relationships remained when evaluating live birth/ongoing pregnancy per cycle. In intrauterine insemination cycles, initial strict morphology was associated with subsequent fecundability only when inseminating total motile count was below 5 million. For cycles with total motile count above this threshold, no impact of low morphology on success rates with intrauterine insemination was observed.

## Introduction

Male factor infertility is a significant contributor to infertility in up to 40% of couples who present for care [1]. The semen analysis is a useful tool to assess the male partner for subfertility, but is imperfect with a significant overlap in sperm parameters between fertile and infertile populations of men [2]. As an assessment tool, strict morphology was originally used to identify couples who would benefit from intracytoplasmic sperm injection (ICSI) when utilizing assisted reproductive technologies. Subsequently it has been studied and identified as a parameter to predict successful outcomes in couples undergoing intrauterine insemination (IUI). Varying thresholds have been reported in the range of 4% to 14%, below which a lower success rate may be expected in couples undergoing IUI [2,3,4], leading to a recommendation to proceed immediately to in vitro fertilization (IVF). However, the data are contradictory, and a systematic review and meta-analysis by Kohn *et. al*. [5] found no clinical difference in IUI pregnancy success among men with normal and abnormal sperm morphology when accounting for total motile sperm count (TMC) and female age.

For many infertile couples, the cost of IVF may be prohibitively expensive. With several different threshold values reported in the literature, evidence-based treatment decisions for couples with low strict morphology may be challenging. The primary aim of this study was to compare pregnancy rates for infertile couples undergoing IUI with various strict morphology values on initial semen analysis. The objective of this research was to determine whether IUI with the partner’s sperm is a reasonable option once low strict morphology is identified on semen analysis during the fertility evaluation, given the significant cost disparity between IUI and IVF. Secondary objectives were to evaluate whether patient or cycle characteristics, specifically female age and inseminating TMC, interact with low morphology to impact success rates of IUI.

## Materials and methods

Approval for this study was obtained through the University of Oklahoma Health Sciences Center Institutional Review Board prior to conducting this study. The IRB also provided an exemption from obtaining consent from patients due to the retrospective nature of the data collection. Charts for all couples undergoing IUI between July 1, 2007 and July 30, 2012 at a university-based infertility practice were reviewed. Couples were excluded in the absence of strict morphology data on a semen analysis and for the use of donor sperm. Cycles were excluded if more than one IUI procedure was performed in a given cycle and for missing data such as cycle outcome. Patient characteristics were obtained including the woman’s age, ethnicity, body mass index (BMI), duration of infertility and infertility diagnosis. Semen analysis parameters were noted during the initial infertility evaluation including strict morphology. The characteristics of each IUI cycle were reviewed including medications used for ovulation induction or ovarian stimulation as well as the TMC in the inseminating sample for IUI. After data collection, the database was anonymized before data analysis was undertaken. The primary outcome was positive pregnancy test per cycle, defined as a serum quantitative human chorionic gonadotropin (hCG) > 10 mIU/mL fifteen days following IUI. The secondary outcome was live birth/continuing pregnancy. Live birth was defined as delivery of a viable infant, and continuing pregnancy was defined as two ultrasounds in the first trimester documenting fetal heart beat and appropriate interval growth.

Semen specimens from male partners were collected at the clinic or offsite by masturbation. In the case of off-site collections, semen specimens were taken to the andrology lab for processing within an hour of collection and maintained at body temperature during transport. Following collection, the volume of the specimen was recorded using a graduated pipette and the sample mixed and allowed to undergo liquefaction after which a 7ul aliquot was placed in a pre-warmed MicroCell (Vitrolife) counting chamber and assessed for concentration, motility and progression. Motility (%) was measured by manually counting at least 200 cells. Sperm TMC was calculated according to the formula “volume x count x motility.” Strict morphology was assessed on samples provided for semen analysis. For these samples, 5ul of the semen was applied to a clean microscope slide, spread in a thin layer and allowed to air dry. The slide was then stained (STAT III andrology stain, Mid-Atlantic Diagnostics) and 100 cells were evaluated for strict morphology according to previously established criteria [6–11]. All laboratory personnel were trained in the performance of semen analysis and participated in internal and external quality control programs per the Clinical Laboratory Improvement Amendments (CLIA) guidelines.

Semen samples for IUI cycles were processed according to the following protocol; following liquefaction the sample volume, count and progression was recorded. If the specimen had a TMC of ≥  20 x 10^6^ a single phase density gradient was used in which semen was gently layered on a bed of pre-warmed 90% gradient solution (Sperm Care, In Vitro Care, Inc.) in 1 or 2 tubes, depending upon the semen volume. Centrifugation was performed at 400 x g for 20 minutes at room temperature (RT) or until a pellet formed. A glass Pasteur pipette was used to carefully remove the supernatant down to the pellet which was then re-suspended in 3.0 ml of fresh sperm washing medium (SWM; HTF Hepes +  5.0 mg/ml HSA, In Vitro Care) and centrifuged for 10 minutes at 400 x g at RT. After the second wash the supernatant was removed and the pellet re-suspended in 0.5 ml of SWM and mixed thoroughly. Seven ul of the sperm suspension was evaluated for count, motility, and progression and the TMC determined post-processing after which the specimen was used for insemination. In cases where the initial TMC was ≤  20 x 10^6^, specimens were subjected to wash only. This cutoff was established in our clinical practice based on an expected recovery of 25% of motile sperm following density gradient centrifugation, which would provide approximately 5.0 x 10^6^ motile sperm for insemination. In this case specimens were diluted with SWM in a 2:1 ratio (SWM/Semen) and mixed thoroughly using a serological pipette. Specimens were then centrifuged at 400 x g for 10 minutes at RT. Following the initial wash, the supernatant was removed, the pellet re-suspended in 3.0 ml of SWM and the wash step repeated. The supernatant was removed and the pellet re-suspended in 0.5 ml of SWM and mixed thoroughly. Seven ul of the sperm suspension was evaluated for count, motility, and progression and the TMC determined post-processing after which the specimen was used for insemination.

Couples were categorized by strict morphology ≤ 1%, 2-4%, 5-8%, 9-14%, and > 14% as reported on their initial semen analysis at the time of infertility evaluation. Chi-square tests and Fisher’s exact tests were used to evaluate the distribution of baseline patient characteristics and pregnancy results by strict morphology. Couple characteristics including female age, female BMI, duration of infertility in years, and TMC of sample inseminated were compared among strict morphology categories using Kruskal-Wallis tests for non-normal distributions. Data from all IUI cycles were analyzed using a generalized estimating equations (GEE) methodology to fit a Poisson regression model with robust standard errors to account for correlation of multiple IUI treatment cycles within the same couple (cluster). The models included pregnancy as the dependent variable and categorical strict morphology as the independent variable of interest using > 14% normal morphology as the reference group. To address the informative cluster size, which may occur when the number of IUI cycles per couple is influenced by previous treatment outcomes, a cluster-weighted model was fit by weighting the GEE score equation by the inverse of the number of IUI cycles completed for each couple [12,13]. Risk ratios (RR) and 95% confidence intervals (95% CI) are reported. Covariates evaluated as potential confounders included female age (continuous), race/ethnicity (Caucasian, Hispanic, American Indian, Asian and Black), BMI (<25, 25-29.9, ≥  30 kg/m^2^), duration of couple’s infertility (<3 vs. ≥  3 years), female partner’s infertility diagnosis (none vs. ovulatory, tubal, endometriosis or other), medication used for ovulation induction or ovarian stimulation (none, clomiphene citrate/letrozole, gonadotropins), and sperm TMC (<5, 5-20, > 20 million) at the time of IUI (post washing). Confounding was defined as >  15% change in the measures of association for strict morphology when comparing unadjusted and adjusted models. Potential modification of strict morphology associations by inseminating TMC (at time of IUI after sperm washing) or female diagnosis was assessed by adding interaction terms to the model. Categories of strict morphology were collapsed to ≤  4 and > 4 for assessment of interactions due to cells with sparse data. *P*-values < 0.05 were considered statistically significant. Statistical analyses were performed using SAS 9.4 software (SAS Institute Inc, Cary NC).

## Results

During the study period, 2221 IUI cycles were performed on 719 couples. Of these, 274 cycles were excluded from the analysis due to the use of donor sperm and 517 did not have strict morphology determination performed on semen analysis prior to initiating treatment. Twenty-eight cycles were excluded for reasons including: a) two samples collected and combined for IUI (n = 15), b) two IUIs in the same treatment cycle (n = 4), c) the details of the IUI procedure were not documented (n = 2), or d) partner-reported sample spill (n = 4). No cases of retrograde ejaculation were identified for exclusion among the remaining cycles. Cycles with missing covariate information (n = 82) or missing pregnancy outcome (n = 33) were excluded. Charts remaining for analysis included 1287 IUI cycles performed on 435 couples. The distribution of patient characteristics did not differ by strict morphology when comparing Caucasians and non-Caucasians, normal weight and overweight/obese women, primary and secondary infertility, those with or without endometriosis, ovulatory, tubal, or other infertility diagnoses or those using medications for ovulation induction (Table 1). Low strict morphology ( ≤ 4%) was present more frequently when the inseminating TMC was <  5 million and when duration of infertility exceeded 3 years. Median inseminating TMC was significantly higher among couples with > 14% normal morphology, while the female partner’s median age (p = 0.0499) was significantly lower in this group (Table 2).

**Table 1:** Distribution of characteristics by strict morphology for 435 couples receiving intrauterine insemination^a^.

% Normal morphology	≤1% n (%)	2-4% n (%)	5-8% n (%)	9-14% n (%)	≥14% n (%)	*p* ^b^
**Female Race**						0.36
Caucasian	13 (3.7)	76 (21.7)	139 (39.6)	93 (26.5)	30 (8.6)	
Non-Caucasian	4 (4.8)	11 (13.1)	37 (44.1)	27 (32.1)	5 (6.0)	
**Female Age**						0.09
≥ 35 years	4 (3.5)	20 (17.4)	44 (38.3)	42 (36.5)	5 (4.4)	
< 35 years	13 (4.1)	67 (20.9)	132 (41.3)	78 (24.4)	30 (9.4)	
**Female BMI (kg/m**^2^)						0.23
≥ 25	11 (4.6)	44 (18.6)	89 (37.6)	75 (31.7)	18 (7.6)	
< 25	6 (3.0)	43 (21.7)	87 (43.9)	45 (22.7)	17 (8.6)	
**Years of Infertility**						0.06
≥ 3 years	12 (7.1)	42 (23.1)	71 (39.0)	43 (23.6)	14 (7.7)	
<3 years	5 (1.6)	45 (17.8)	105 (41.5)	77 (30.4)	21 (8.3)	
**Total Motile Sperm Count (x 10**^**6**^)^d^						<0.0001
< 5	7 (8.6)	27 (33.3)	28 (34.6)	19 (23.5)	0 (0.0)	
5-20	8 (4.1)	37 (18.8)	84 (42.6)	55 (27.9)	13 (6.6)	
> 20	2 (1.3)	23 (14.7)	64 (40.8)	46 (29.3)	22 (14.0)	
**Type of Infertility**						0.29
Primary	11 (3.2)	67 (19.4)	147 (42.6)	94 (27.3)	26 (7.5)	
Secondary	6 (6.7)	20 (22.2)	29 (32.2)	26 (28.9)	9 (10.0)	
**Endometriosis**						0.43
Yes	3 (6.0)	8 (16.0)	18 (36.0)	14 (28.0)	7 (14.0)	
No	14 (3.6)	79 (20.5)	158 (41.0)	106 (27.5)	28 (7.3)	
**Ovulatory Diagnosis**						0.28
Yes	4 (2.4)	33 (20.1)	71 (43.3)	39 (23.8)	17 (10.4)	
No	13 (4.8)	54 (19.9)	105 (38.8)	81 (29.9)	18 (6.6)	
**Tubal Diagnosis**						0.83^c^
Yes	1 (5.3)	3 (15.8)	9 (47.4)	4 (21.1)	2 (10.5)	
No	16 (3.9)	84 (20.2)	167 (40.1)	116 (27.9)	33 (7.9)	
**Other Diagnosis**						0.11
Yes	5 (9.8)	11 (21.6)	17 (33.3)	16 (31.4)	2 (3.9)	
No	12 (3.1)	76 (19.8)	159 (41.4)	104 (27.1)	33 (8.6)	
**Medications for ovulation induction**						0.67^c^
None (natural)	0 (0.0)	4 (28.6)	6 (42.9)	3 (21.4)	1 (7.1)	
Clomiphene/Letrozole	16 (4.2)	77 (20.2)	158 (41.4)	102 (26.7)	29 (7.6)	
Gonadotropins	1 (2.6)	6 (15.4)	12 (30.8)	15 (38.5)	5 (12.8)	

^a^Characteristics reported at the initial clinical visit;

^b^Chi-square test, unless otherwise noted;

^c^Fisher’s exact test;

^d^At post-wash at initial IUI.

**Table 2:** Median (25^th^ and 75^th^ percentiles) female age, body mass index, infertility duration and total motility by strict morphology categories for 435 couples receiving intrauterine insemination^a^.

% Normal morphology	≤1% (n = 17) Median (25^th^, 75^th^)	2-4% (n = 87) Median (25^th^, 75^th^)	5-8% (n = 176) Median (25^th^, 75^th^)	9-14% (n = 120) Median (25^th^, 75^th^)	>14% (n = 35) Median (25^th^, 75^th^)	*p* ^b^
Age (years)	30.0 (30.0,32.0)	31.0 (27.0,34.0)	31.0 (28.0,34.5)	32.0 (29.0,36.0)	29.0 (26.0,32.0)	0.05
BMI (kg/m^2^)	29.0 (23.1,32.9)	25.3 (21.4,31.8)	25.1 (22.0,32.0)	25.9 (22.4,30.8)	25.6 (22.1,29.9)	0.73
Infertility duration (yrs)	3.5 (2.3,4.0)	2.5 (1.5,4.0)	2.5 (1.5,3.5)	2.0 (1.4,3.5)	2.3 (1.5,3.5)	0.16
Total Motile Count inseminated^c^	5.8 (2.2,10.6)	9.1 (4.6,21.3)	14.0 (6.4,26.8)	15.1 (6.9,30.9)	37.2 (16.5,67.6)	<0.0001

^a^Characteristics reported at the initial clinical visit

^b^Kruskal-Wallis test;

^c^Total motile count assess at time of insemination

The pregnancy rate among all IUI cycles was 14.4% (185/1287 cycles). In unadjusted models, no associations were observed between strict morphology on initial semen analysis and pregnancy in subsequent IUI cycles (Table 3). When entering the covariates into the model individually to evaluate confounding, only inseminating TMC met the previously specified criteria to be considered a confounder. When inseminating TMC was controlled in the analysis, the adjusted risk ratios for strict morphology categories increased in magnitude but remained near unity and all 95% confidence intervals included the null value (Table 3). For couples with a low pre-treatment morphology, the fecundability among IUI cycles did not differ from that of couples with normal morphology above the threshold of > 14% [RR for ≤ 1%: 1.22 (95% CI 0.51-2.93) and RR for 2-4%: 1.00 (RR 1.00 95% CI 0.53 – 1.91)]. Live birth/ongoing pregnancy rates also did not differ in any morphology category compared to the referent group in the unadjusted and adjusted models (Table 3).

**Table 3:** Risk ratios and 95% confidence intervals for the association between strict morphology and pregnancy or live birth/continuing pregnancy following intrauterine insemination.

% Normal morphology	# Cycles	Pregnancy (%)	Unadjusted RR^a^	95% CI	Adjusted RR^a^^,^^b^	95% CI
≤1	45	7 (15.6%)	0.99	0.41–2.40	1.22	0.51–2.93
2–4	253	38 (15.0%)	0.90	0.48–1.70	1.00	0.53–1.91
5–8	523	78 (14.9%)	1.06	0.59–1.88	1.13	0.63–2.01
9–14	352	45 (12.8%)	1.01	0.54–1.86	1.07	0.58–1.97
>14	114	17 (14.9%)	1.00	Reference	1.00	Reference
% Normal morphology	# Cycles	Live birth/ Continuing Pregnancy (%)	Unadjusted RR^a^	95% CI	Adjusted RR^a^^,^^b^	95% CI
≤1	45	5 (11.1%)	1.00	0.33-3.06	1.34	0.44-4.11
2–4	253	27 (10.7%)	0.98	0.45-2.13	1.14	0.52-2.50
5–8	523	50 (9.6%)	1.12	0.54-2.29	1.22	0.60-2.49
9–14	352	30 (8.5%)	0.95	0.44-2.06	1.04	0.48-2.22
>14	114	11 (9.7%)	1.00	Reference	1.00	Reference

RR = risk ratios; CI = confidence intervals;

^a^Cluster-weighted Poisson regression model with robust standard errors used to calculate risk ratios and 95% confidence intervals

^b^Regression model adjusted for intrauterine insemination sperm total motile count.

Compared to inseminating TMC >  20 million, inseminating TMC <  5 million was associated with a reduced pregnancy rate approaching borderline statistical significance (TMC <  5 million: RR = 0.61, 95% CI 0.36-1.03; TMC 5-20: RR = 0.96, 95% CI 0.69-1.33, adjusted for strict morphology). When examining interactions between strict morphology and inseminating TMC, cycles with a combination of low morphology ≤  4% and TMC inseminated < 5 million were 63% less likely to have a positive pregnancy test following IUI when compared to those with higher morphology (>4%) and TMC inseminated > 20 (0.37, 95% CI 0.17-0.82; Table 4). Low morphology ( ≤ 4 vs > 4) did not reduce pregnancy rates when couples had inseminating TMC of 5-20 or > 20 million. Similarly, the TMC-adjusted association with low morphology did not differ among couples with (n = 751, RR = 0.94, 95% CI 0.55-1.62) and without (n = 536, RR = 0.94, 95% CI 0.56-1.58) female infertility diagnoses. The conclusions were unchanged when associations were examined separately within each non-mutually exclusive diagnostic category (endometriosis RR = 1.50, 95% CI 0.39-5.84; tubal RR = 2.58, 95% CI 0.74-8.96); ovulatory RR = 0.67, 95% CI 0.37-1.22; and “other” diagnosis RR = 1.83, 95% CI 0.57-5.92). Results were consistent when associations with live birth/continuing pregnancies were examined (Table 4).

**Table 4:** Unadjusted risk ratios and 95% confidence intervals for the association between strict morphology and intrauterine insemination outcomes stratified by inseminating total motile sperm count.

	Inseminating total motile sperm count (million)					
** < 5**		**5–20**		** > 20**	
Cycles	RR^a^ (95% CI)	Cycles	RR^a^ (95% CI)	Cycles	RR^a^ (95% CI)
**Pregnancy**						
Normal morphology ≤ 4%	83	0.37 (0.17-0.82)	158	1.00 (0.61-1.64)	57	1.21 (0.62-2.34)
> 4%	142	0.81 (0.46-1.41)	487	0.99 (0.69-1.44)	360	1.00
**Live birth/continuing pregnancy**						
Normal Morphology ≤ 4%	83	0.32 (0.11-0.92)	158	1.07 (0.60-1.89)	57	1.24 (0.56-2.79)
> 4%	142	0.62 (0.28-1.34)	487	0.99 (0.63-1.55)	360	1.00

RR =  risk ratios; CI =  confidence intervals

^a^Cluster-weighted Poisson regression model with robust standard errors used to calculate unadjusted risk ratios

## Discussion

Our study did not demonstrate a significant relationship between morphology and fecundability in IUI treatments overall, but did observe reduced pregnancy and live birth/continuing pregnancy rates when couples experienced both low morphology and inseminating TMC below 5 million. While these results contrast with some prior studies indicating higher threshold values for a reasonable expectation of success with IUI, they do agree with other studies reporting the importance of considering the combined effect of low morphology and low TMC on pregnancy rates. This information adds to our current knowledge of IUI success rates and can be utilized when counseling patients about treatment options.

Strict morphology was developed initially as a parameter to predict successful IVF outcomes [6], and was subsequently shown to be of prognostic value in IUI cycles in some studies [14–20]. There is recognized inter- and intra-laboratory variation in the reporting of morphology as a semen parameter [21,22]. Drift toward stricter criteria within a single laboratory over time has been described [23]. Over the last few decades, the number of morphologically normal spermatozoa has diminished due to methodological changes in the way that strict morphology is determined [24], with 14% normal forms initially considered normal [3] and 4% considered normal by the 2010 WHO criteria [4]. This phenomenon has increased the number of male partners diagnosed with teratozoospermia, and as a result morphology has become less predictive of pregnancy outcomes over time. A systematic review and meta-analysis did not demonstrate a relationship between morphology alone and IUI success and likely reflects this change over time [5].

Our retrospective study included multiple cycles for a number of included couples. Because these cycles therefore cannot be considered independent data points, we used statistical methods to account for multiple measures. We report live birth and ongoing pregnancy combined as the secondary outcome, representing an approximation of total live birth. Although some couples were lost to follow up after viable clinical intrauterine pregnancy was established by ultrasound, we included them in this composite outcome because the pregnancy was still ongoing at the point of last interaction. Although this incomplete data on live birth outcomes is a potential limitation of our study, miscarriage rates are low after 9-10 weeks of gestation, making ongoing pregnancy a reasonable approximation of the number of couples who would go on to deliver a live birth [25,26].

In a recent secondary analysis of a randomized controlled trial, inseminating total motile sperm count has been identified as a significant contributor to IUI success rates [27], and our finding that low inseminating TMC negatively affects pregnancy rates within morphology categories agrees with previous data. An increasing number of abnormal parameters on the semen analysis corresponds to an increasing risk of infertility among males, and so within the infertility population a number of male patients can be expected to have co-occurring low motility and low morphology [2]. Low motility on the initial semen analysis is anticipated to result in low TMC in the inseminating semen sample, so it is important to investigate the relationship between TMC and low morphology, as we have done. Semen samples for insemination in this study were processed by two different methods based on clinical protocols, but this has not been shown to make a difference in live birth rates in IUI cycles [27] and we would not expect this to affect our conclusions. An important limitation of this analysis was that multiple characteristics of the male partner (smoking, obesity, age) were not collected in the database and so confounding due to these unmeasured factors cannot be excluded. Additionally, because of the retrospective design, many semen parameter assessments in this study were performed prior to release of the updated WHO 2010 guidelines, so should be interpreted with this in mind. Confirming these findings in a larger data set such as a national database would strengthen the conclusions, although within the United States IUI cycles are not tracked in a similar manner to assisted reproductive technology (ART) making access to a larger data set outside of randomized clinical trials more challenging.

This study classifies couples into morphology category based on the initial semen analysis completed during the fertility workup, reflecting typical clinical practice and increasing generalizability of our results. This is the morphology measurement that will be available to the provider when the decision whether or not to proceed to IUI is made, and is therefore the most important morphology value. This improves the generalizability of our findings to routine care settings. We also categorize couples into a very low morphology category when morphology is 1% or less, the group most likely to be counseled to proceed directly to IVF rather than attempting less invasive treatment such as IUI, and even in this group we were able to show similar pregnancy rates to couples with normal morphology. For many couples in treatment in the United States, insurance does not cover fertility treatment and having low cost options can make the difference in ability to proceed with treatment.

In conclusion, unless the inseminating TMC is below 5 million, based on the findings in this study, IUI may be reasonable as a first line treatment for couples with low morphology identified in the initial semen analysis during their fertility workup, in the absence of other indications for IVF.

## Supporting information

S1 File Data codebook clean. (DOCX)

S2 File Study data. (XLSX)
