# Supplementary material for: Low morphology does not lower success after intrauterine insemination unless inseminating motile sperm count is low
Source: PLoS One. 2025 Mar 19;20(3):e0317521. doi: 10.1371/journal.pone.0317521 (PMC11922279; doi:10.1371/journal.pone.0317521)
Supplement: S1 File — (DOCX) [file pone.0317521.s001.docx]

**Morphology Database [Burks et al. PLOS ONE]**

Contains records for 1,287 intrauterine insemination cycles among 435 patients

| **#** | **Variable Name** | **Type** | **Description** | **Values** |
| --- | --- | --- | --- | --- |
| **1** | study_id | Numeric | Study ID | Numeric study ID |
| **2** | morphology | Numeric (categorical) | Strict morphology at infertility evaluation | 1: ≤1% normal  2: 2-4% normal  3: 5-8% normal  4:9-14% normal  5: >14% normal |
| **3** | morph_binary | Numeric (binary) | Strick morphology at infertility evaluation (at 4% normal or below) | 0: > 4 % normal  1: ≤ 4 % normal |
| **4** | tot_motile | Numeric (continuous) | Total motile sperm count at insemination (defined as volume x count x motility) | Numeric value (millions) |
| **5** | tmc_cat | Numeric (categorical) | Total motile sperm count at insemination (post washing) | 1: < 5 million  2: 5-20 million  3: >20 million |
| **6** | delivery_contpreg | Numeric (binary) | Live birth or ongoing pregnancy (defined as two ultrasounds in the first trimester documenting fetal heart beat and appropriate interval growth) | 0: No  1: Yes |
| **7** | preg | Numeric (binary) | Positive pregnancy test (defined as serum quantitative human chorionic gonadotropin (hCG) >10 mIU/mL fifteen days following IUI) | 0: No  1: Yes |
| **8** | age | Numeric (continuous) | Female age (years) | Years |
| **9** | agecat | Numeric (binary) | Female age (over 35 years) | 0: < 35 years  1: ≥ 35 years |
| **10** | race_ethnicity | Numeric (Categorical) | Race/ethnicity | 1: Non-Hispanic White  2: Hispanic  3: American Indian  4: Asian  5: Black |
| **11** | NHWhite | Numeric (binary) | Non-Hispanic White race/ethnicity | 0: No  1: Yes |
| **12** | BMI | Numeric (Continuous) | Female body mass index (kg/m^2^) | Kg/m^2^ |
| **13** | OW_obese | Numeric  (Categorical) | Female overweight or obese | 0: BMI <25  1: BMI ≥ 25 |
| **14** | yrs_infertile | Numeric (continuous) | Duration of infertility (years) | years |
| **15** | infert_3yrs | Numeric (binary) | Duration of infertility (3 or more years) | 0: <3 years  1: ≥ 3 years |
| **16** | dx_Female | Numeric (binary) | Female partner’s infertility diagnosis | 0: None  1: Ovulatory, tubal, endometriosis or other diagnosis |
| **17** | dx_endo | Numeric (binary) | Endometriosis | 0: No  1: Yes |
| **18** | dx_ovulate | Numeric (binary) | Ovulatory diagnosis | 0: No  1: Yes |
| **19** | dx_tubal | Numeric (binary) | Tubal diagnosis | 0: No  1: Yes |
| **20** | dx_other | Numeric (binary) | Other female partner diagnosis | 0: No  1: Yes |
| **21** | medication | Numeric (categorical) | Medication used for ovulation induction or ovarian stimulation | 0:None  1: clomiphene citrate or letrozole  2: gonadotropins |
| **22** | count | Numeric (ordinal) | Number of IUI cycles completed (number of completed cycles may exceed number of records in analytic database due to excluded cycles) | Count |
| **23** | inverse_count | Numeric  (ordinal) | Inverse of IUI cycle count (used for weighting) | 1/Count |
| **24** | primary | Numeric (binary) | Primary or secondary infertility | 0: Secondary infertility  1: Primary infertility |
| **25** | firstobs | Numeric (binary) | Initial visit (first observation) | 0: No  1: Yes |
